# Bamboo Shoot and *Artemisia capillaris* Extract Mixture Ameliorates Dextran Sodium Sulfate-Induced Colitis

**DOI:** 10.3390/cimb44100345

**Published:** 2022-10-20

**Authors:** Hee-Jun Kim, Bohye Kim, Mi-Ra Lee, Moonjin Ra, Yongjun Lee

**Affiliations:** Hongcheon Institute of Medicinal Herb, Hongcheon-gun 25142, Korea

**Keywords:** colitis, inflammatory bowel disease, bamboo shoot, *Artemisia capillaris*, dextran sodium sulfate

## Abstract

Inflammatory bowel disease (IBD) is a chronic inflammatory disease of the gastrointestinal tract and is characterized by recurrent chronic inflammation and mucosal damage of the gastrointestinal tract. Recent studies have demonstrated that bamboo shoot (BS) and *Artemisia capillaris* (AC) extracts enhance anti-inflammatory effects in various disease models. However, it is uncertain whether there is a synergistic protective effect of BS and AC in dextran sodium sulfate (DSS)-induced colitis. In the current study, we tested the combined effects of BS and AC extracts (BA) on colitis using in vivo and in vitro models. Compared with control mice, oral administration of DSS exacerbated colon length and increased the disease activity index (DAI) and histological damage. In DSS-induced colitis, treatment with BA significantly alleviated DSS-induced symptoms such as colon shortening, DAI, histological damage, and colonic pro-inflammatory marker expression compared to single extracts (BS or AC) treatment. Furthermore, we found BA treatment attenuated the ROS generation, F-actin formation, and RhoA activity compared with the single extract (BS or AC) treatment in DSS-treated cell lines. Collectively, these findings suggest that BA treatment has a positive synergistic protective effect on colonic inflammation compared with single extracts, it may be a highly effective complementary natural extract mixture for the prevention or treatment of IBD.

## 1. Introduction

Inflammatory bowel disease (IBD) is a chronic inflammatory digestive disease characterized by gastrointestinal tract inflammation and is mainly composed of two main multifactorial diseases: Crohn’s disease and ulcerative colitis [1,2]. Worldwide, the prevalence of IBD is approximately 396 cases per million people annually; the incidence of IBD is 5 to 245 cases per million people for ulcerative colitis and 1 to 160 cases per million people for Crohn’s disease [3]. The pathological and clinical characteristics of IBD are inflammation of the mucosa of the intestinal tract, which can be exacerbated by ineffective epithelial barrier function, environmental factors, genetic susceptibility, or impaired immune response to the gut microbiota. Furthermore, infection, loss of blood supply, and invasion of the colon wall with collagen or lymphocytic cells are the causes of colonic inflammation [4]. Although the pathogenesis of IBD has not been fully clarified yet, it is generally accepted that the cause of IBD is multifactorial, including genetic predisposition, gastrointestinal microbiota disorders, and altered dysregulated immune responses [5,6,7]. This study aimed to investigate a therapeutic approach to IBD prevention and therapeutics, which could soften patient outcomes and be efficacious, less toxic, and cost-effective.

Bamboo shoots (BS) contain high contents of proteins, carbohydrates, essential amino acids, vitamins, fibers, and minerals, and low fat. In addition, BS has numerous health benefits, including modulated body weight, improved digestive system, and decreased cholesterol levels [8,9]. In particular, BS and bamboo shoot shells of *Pleioblastus amarus* exhibit anti-inflammatory effects through the inhibition of ERK signaling [10]. BS extract also exerts inhibitory activity on LPS-induced inflammation by manipulating AMPK signaling [11]. Bamboo shoot dietary fiber-1(BSDF-1) has protective effects and regulates anti-inflammatory activity by inactivating inflammatory signaling pathways, including nuclear factor-kappa B (NF-κB) and NOD-like receptor pyrin domain-containing protein 3 (NLRP3) inflammasomes in DSS-induced colitis. Furthermore, the BSDF-1 administration significantly decreased pathological colonic damages (disruption of the architecture of colonic mucosa and ulceration) and recovered the mRNA expression of tight junction proteins, such as zonula occludens-1 (ZO-1), claudin-1, and occludin [12]. 

*Artemisia capillaris* (AC) is a traditional oriental medicinal herb with various pharmacological activities, including hepatoprotective, cholestasis, analgesic, and antipyretic [13,14,15]. AC contains a variety of flavonoids, phenolic acids, terpenoids, coumarins, chlorogenic acid, ascorbic acid, scoparone, and essential oil, which promotes anti-oxidant, anti-inflammatory, anti-steatotic, anti-viral, and anti-tumor effects [16]. In particular, a recent study demonstrated that AC treatment ameliorated the severity of trinitrozobenzoic acid (TNBS)-induced colitis by inhibiting reactive oxygen species (ROS) generation and downregulating pro-inflammatory signaling pathways such as STAT3/MAPK/NF-κB [17]. However, the potential synergistic effect of BA in DSS-induced colitis is uncertain.

Ras homolog gene family, member A (RhoA)/Rho-associated coiled-coil forming protein kinase controls various functions in the regulation of cytoskeleton organization, membrane trafficking, cellular morphology, motility, polarity, cell division, and cell cycle progression [18]. RhoA activity is regulated by cycling between an inactive GDP-bound form and an active GTP-bound form. RhoA is activated by interacting with guanine nucleotide exchange factors (GEFs), which catalyze the exchange of GDP for GTP. In contrast, GTPase-activating proteins (GAPs), stimulate the intrinsic GTP hydrolysis (GTPase) reaction [18,19,20]. In addition, RhoA/ROCK activity plays a key regulator role in multiple types of solid organ fibrosis, including intestinal fibrosis [21]. In colitis models, inhibition of RhoA/ROCK activity significantly protects the intestinal epithelial barrier dysfunction and colonic inflammation via STAT3 and NF-κB signaling pathways [22,23,24,25]. It is known that STAT3-NF-κB and RhoA/ROCK signaling pathway is closely correlated with epithelial barrier dysfunction and conical inflammation in the colitis model.

In this study, we investigated the potential synergistic effect of BA in DSS-induced colitis. This effect was mediated by downregulating the NF-κB-STAT3 signaling pathway and RhoA/ROCK activation, which controls colonic inflammation and F-actin polymerization and plays an important role in the pathogenesis of colitis.

## 2. Materials and Methods

### 2.1. Materials

BS and AC extracts were provided by CHAMMAC Co., Ltd. (Goyang, Korea). Dextran sulfate sodium (DSS MW 36,000–50,000) was purchased from MP Biomedicals (Solon, OH, USA). The anti-phospho-STAT3 (Tyr705), anti-STAT3, anti-phospho-p65 (Ser536), anti-p65, and anti-cyclooxygenase-2 (COX-2) antibodies were obtained from Cell Signaling Technology (Danvers, MA, USA). Anti-glyceraldehyde-3-phosphate dehydrogenase (GAPDH) antibody was obtained from Santa Cruz Biotechnology (Santa Cruz, CA, USA). Anti-inducible nitric oxide synthase (iNOS) antibody was purchased from Merck Millipore (Lake Placid, NY, USA).

### 2.2. Cell Line Culture

Human colorectal adenocarcinoma cell lines, HT-29, and Caco2 cell lines were obtained from the Korean Cell Live Bank (30038 and 30037.1, Seoul, Korea). HT-29 cells were grown in Roswell Park Memorial Institute (RPMI) 1640 supplemented with 300 mg/L L-glutamine, 25 mM HEPES, and 25 mM NaHCO_3_, 10% heat-inactivated fetal bovine serum (FBS; Thermo Fisher Scientific, Waltham, MA, USA), 100 units/mL penicillin, and 100 µg/mL streptomycin (Thermo Fisher Scientific) and maintained in an incubator, under a humidified atmosphere with 5% CO_2_ and 37 °C. HT-29 cells were subcultured once every 5 days at 70–80% confluence by treatment with 0.5% trypsin–EDTA (Thermo Fisher Scientific). Caco2 cells were cultured in Dulbecco’s modified Eagle’s minimum essential medium (DMEM, Thermo Fisher Scientific) supplemented with 4.5 g/L glucose, 4 mM L-glutamine, 110 mg/mL sodium pyruvate, 10% heat-inactivated FBS, 100 units/mL penicillin, and 100 µg/mL streptomycin. Cells were maintained at 37 °C with 5% CO_2_ in a humidified atmosphere and routinely sub-cultured once a week at 70–80% confluence by treatment with 0.5% trypsin–EDTA (Thermo Fisher Scientific). The culture medium was refreshed every other day. For experiment, HT-29 and Caco2 cells were incubated with 2% DSS (MP Biomedicals) in presence or absence of 200 µg/mL BA extract mixtures [BS (BS:AC, 100:0), AC (BS:AC, 0:100), and BA (BS:AC, 50:50)] for 24 h. HT-29 and Caco2 cells were washed and harvested (CON, DSS, and DSS with BA extract mixture (BS, AC, and BA). Cells were used for Western blot analysis, NF-κB phosphorylation detection, ROS generation, F-actin polymerization, and RhoA activation assay.

### 2.3. Animal Experimental Design

Male C57BL/6N mice (7 weeks old, 20–22 g, *n* = 70) were purchased from Dae Han Bio Link Co., Ltd. (Eumseong, Korea) and housed in a clean facility under natural light-dark cycle conditions (room temperature 22 ± 1 °C, relative humidity at 50–60%, 12 h/12 h light/dark cycle) with clean water and the Teklad Global 18% Protein Rodent Diet (2918C, ENVIGO, Indianapolis, IN, USA). After 1 week of acclimation, the mice were randomly divided into seven groups (*n* = 10 per group). From days 1 to 5, the seven different groups of mice were orally treated the following: control group (CON) saline; DSS group (DSS) 2.5% DSS with saline; BA (BS:AC, 0:100) group, 2.5% DSS with 250 mg/kg AC; BA (BS:AC, 30:70) group, 2.5% DSS with 250 mg/kg BS:AC (30:70); BA (BS:AC, 50:50) group, 2.5% DSS with 250 mg/kg BS:AC (50:50); BA (BS:AC, 70:30) group, 2.5% DSS with 250 mg/kg BS:AC (70:30); and BA (BS:AC, 100:0) group, 2.5% DSS with 250 mg/kg BS, daily. BS and AC were dissolved in saline. All experiments were performed in accordance with Korean laws and with the approval of the Hongcheon Institute of Medicinal Herb Animal Care and Use Committee (HIMH A21-04).

### 2.4. Induction of Colitis and BA Treatment

The DSS-induced ulcerative colitis mouse model was generated as previously described [26]. Colitis was induced in C57BL/6N mice with 2.5% DSS (molecular weight 36,000–50,000) dissolved in drinking water provided ad libitum for 5 days. The control mice were administered drinking water without DSS. The BS and AC extract mixtures were dissolved in saline and filtered through syringe filters (0.2 μm). The substance was orally administered at a dose of 250 mg/kg body weight once daily at the same time. Control animals received saline orally once daily. General health, body weight, stool consistency, and bloody stool score were monitored once daily. On 3 and 6 days, the mice were anesthetized using Terrel solution (isoflurane, KyongBo Pharmaceutical Co., Ltd., Asan, Korea). Postmortem, the colon tissue (from the proximal to distal end) was removed from mice groups and the colon was used for clinical scoring, histological analysis, and Western blot studies.

### 2.5. Clinical Score

Two investigators blinded to the treatment groups determined the presence of occult or gross blood per rectum and stool consistency. A scoring system was used to assess diarrhea and the presence of occult or overt blood in the stool. Changes in body weight were indicated as the percentage loss of baseline body weight [27]. Based on the above data, the DAI score was calculated by the sum of three clinical scores (weight loss, stool consistency, and bloody stool) during the DSS treatment period (0–5 days, Table 1).

### 2.6. Histological Analysis

For colon histology, the colon was rolled from the proximal to the distal end [28], fixed in 4% buffered formalin, and embedded in paraffin. Four μm sections were stained with hematoxylin and eosin (H&E) according to standard protocols. Histological scoring was performed in a blinded manner by a pathologist. For cell infiltration, locally increased numbers of inflammatory cells in the lamina propria were scored as 1, the confluence of inflammatory cells extending into the submucosa was 2, and the transmural extension of the infiltrate was 3. For tissue damage, discrete lymphoepithelial lesions were scored as 1, mucosal erosions as 2, and extensive mucosal damage and/or extension through deeper structures of the bowel wall as 3. The two equally weighted subscores (cell infiltration and tissue damage) were added, and the combined histological colitis severity scores ranged from 0 to 6 [29].

### 2.7. Western Blot Analysis

The colonic tissues (from the distal to proximal end) were washed with ice-cold PBS and lysed with a RIPA buffer, Xpert protease inhibitor cocktail, and Xpert phosphatase inhibitor cocktail solution (GenDEPOT, Katy, TX, USA). Tissue homogenates were centrifuged at 15,000× *g* for 15 min at 4 °C, and the protein concentrations in the supernatants were analyzed using a BCA Protein Assay Kit (Thermo Fisher Scientific). Equal amounts of proteins (20 µg/lane) were separated using sodium dodecyl sulfate-polyacrylamide (SDS) gel electrophoresis, transferred onto 0.45 µm pore polyvinylidene fluoride (PVDF) membranes (Merck Millipore, Lake Placid, NY, USA), and blocked with 5% skim milk in 1 × TBS containing 0.1% Tween 20 (TBST) for 1 h at 25 °C. The membranes were incubated with the following primary antibodies overnight at 4 °C: anti-phospho-STAT3 (1:1000, Cell Signaling Technology), anti-STAT3 (1:1000, Cell Signaling Technology), anti-COX-2 (1:1000, Cell Signaling Technology), anti-iNOS (1:1000, Merck Millipore), or anti-GAPDH antibodies (1:5000, Santa Cruz Biotechnology). The membranes were washed with 1×TBST three times for 10 min each and then incubated with the following secondary antibodies for 1 h: goat anti-mouse IgG or goat anti-rabbit IgG (1:5000, Enzo Life Sciences, Farmingdale, NY, USA) at 25 °C. The membranes were then washed with TBST three times for 10 min, and the immunoreactive bands were visualized on digital images captured with a ChemiDoc XRS+ System (BioRad, Hercules, CA, USA) using the EzWestLumi plus western blot detection reagent (ATTO Corporation, Tokyo, Japan). The band intensities were quantified using ImageJ 1.53q software (National institutes of health, Bethesda, MD, USA). Statistical analyses were performed using GraphPad Prism 7 (GraphPad Software, La Jolla, CA, USA).

### 2.8. Detection of NF-κB Phosphorylation in Cultured Cells

HT-29 and Caco2 cells were seeded with a cell number of 3 × 10^5^ per well in 24-well culture plates. After 24 h, cells were cultured and treated as described (Section 2.2). Cells were washed and collected to measure NF-κB phosphorylation using the NF-κB p65 (Total/Phospho) Human InstantOne™ ELISA Kit (Thermo Fisher Scientific, Waltham, MA, USA). An equal volume of the capture antibody reagent and the detection antibody reagent was prepared prior to the experiment. In each well, 50 μL of whole cell lysate, a cell lysis mix (negative control), and a positive control cell lysate were individually added onto the 96-well ELISA plate. Two types of capture antibody reagents, total-NF-κB p65 antibody, and phospho-NF-κB p65 antibody were separately incubated in each well of the pre-coated plate containing sample lysates (50 μL/well) for 1 h. The plate was washed before adding a detection reagent for 30 min and the detection reaction was sequentially inhibited by adding a stop solution (100 μL/well). The absorbance was immediately measured at 450 nm with a correction to 650 nm using an xMark™ microplate absorbance spectrophotometer (BioRad, Hercules, CA, USA). The phospho-NF-κB levels and the ratio of phospho-NF-κB p65 vs. total-NF-κB p65. Data were compared with the control or DSS-treated group. The assays were performed in triplicate.

### 2.9. Measurement of Reactive Oxygen Species Generation

ROS generation was measured using 2′, 7′-dichlorodihydrofluorescein diacetate (DCFH-DA, Sigma-Aldrich, St. Louis, MO, USA). Briefly, DCFH-DA was dissolved in dimethyl sulfoxide (DMSO) and diluted with PBS to a final concentration of 100 μM. HT-29 and Caco2 cells were plated on a 48-well culture plate (1 × 10^5^ per well). After 24 h. Cells were cultured and treated as described (Section 2.2). Cells were washed with PBS three times and then incubated with 100 μM DCFH-DA for 30 min at 37 °C. The loading was terminated by washing the cells with PBS. Accumulation of DCFH-DA in cells was examined by fluorescence microscopy (100×, Axio Observer.D1, Carl Zeiss, Oberkochen, Germany) and measured using a microplate fluorescence reader (SpectraMax GEMINI EM microplate spectrofluorometer, Molecular Devices, Sunnyvale, CA, USA) at 485 nm (excitation wavelengths) and 525 nm (emission wavelengths). The fluorescence intensity was calculated by the relative fluorescence of each treatment compared to control cells. The experiments were performed in triplicate.

### 2.10. RhoA Activation Assay

The RhoA-GTP activity was measured using the G-LISA RhoA activation assay kit (Cytoskeleton Inc., Denver, CO, USA) according to the manufacturer’s instructions. Briefly, HT-29 and Caco2 cells were plated on a 6-well culture plate (1 × 10^6^ per well). After 24 h, cells were cultured and treated as described (Section 2.2). Cells were washed with ice-cold PBS, lysed in 0.3 mL lysis buffer, and placed on ice for 10 min. The cells were then homogenized using a 20-gauge needle for 20 plunges on ice and centrifuged at 14,000× *g* for 5 min at 4 °C. For quantitative detection of active RhoA, absorbance was read at 490 nm using an xMark™ microplate absorbance spectrophotometer (BioRad).

### 2.11. Quantification of Filamentous-Actin (F-Actin) Formation

HT-29 and Caco2 cells were seeded with a cell number of 3 × 10^5^ per well in 24-well culture plates. After 24 h, HT-29 and Caco2 cells were cultured and treated as described (2.2. Cell lines culture). Cells were washed with PBS three times and fixed in 4% paraformaldehyde (methanol-free, Thermo Fisher) for 10 min, permeabilized with 0.1% Triton X-100, washed in PBS, and blocked for 30 min in 1% BSA and 5% goat serum for 1 h at 25 °C. Then, they were rinsed with PBS three times for 10 min each. After the cells were blocked, they were incubated for 30 min at 25 °C with Alexa Fluor 488-conjugated phalloidin (Invitrogen, CA, USA) to visualize F-actin. The samples were examined by fluorescence microscopy (100×, Axio Observer.D1, Carl Zeiss), and the amount of F-actin was determined using a SpectraMax GEMINI EM microplate spectrofluorometer (Molecular Devices) at 485 nm (excitation wavelengths) and 525 nm (emission wavelengths). The fluorescence intensity was calculated by the relative fluorescence of each treatment compared to control cells. The experiments were performed in triplicate.

### 2.12. Statistical Analysis

Statistical analyses were performed, and graphs were generated using GraphPad Prism software (GraphPad Prism 7, GraphPad Software, La Jolla, CA, USA). Statistical differences were determined for colon length, histological scores, band intensities, and fluorescence intensity using one-way analysis of variance (ANOVA) followed by Tukey’s post-hoc test. Two-way ANOVA was used when two parameters were simultaneously compared between distinct treatments (various mixing ratios) and the day after DSS induction using Dunnett’s post-hoc test to determine significant differences between groups. The data are presented as means ± SD. Statistical significance was reached at *p* < 0.05.

## 3. Results

### 3.1. Effects of the Combined Ratio of BA Extracts on Body Weight Loss, Stool Constancy, Bloody Stool, and DAI Score

**Figure 1:**
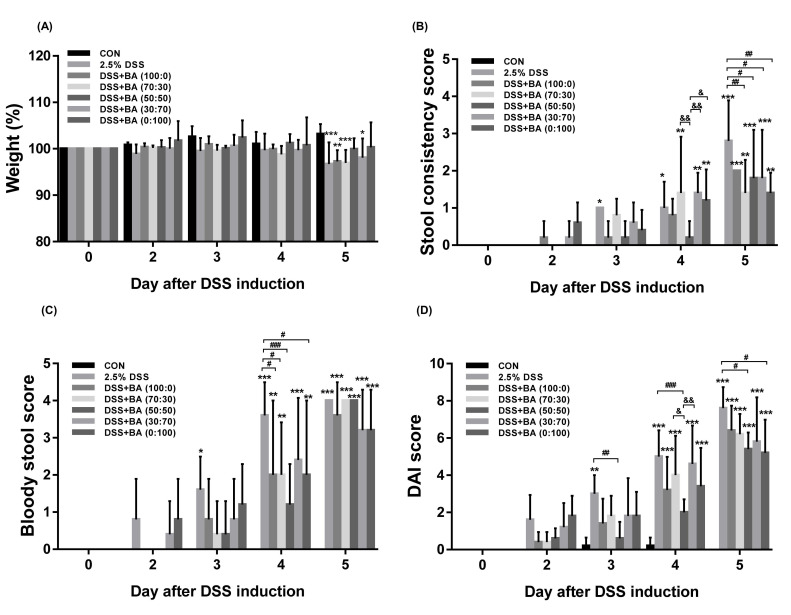
Effects of the combined ratio of BA extract mixtures on body weight loss, stool constancy, bloody stool, and DAI score. C57BL/6N (Male) mice were administrated with 2.5% DSS in drinking water (ad libitum) and treated with or without BA (BS:AC; 0:100, 30:70, 50:50, 70:30, 100:0; 250 mg/kg/day; p.o). (**A**) Body weight, (**B**) Stool consistency score, and (**C**) Bloody stool score was measured daily during the experimental period. (**D**) The disease activity index (DAI) was scored once daily for 5 days. Each data represents the percentage or mean ± SD. Statistical data were obtained by two-way ANOVA with Dunnett’s post-hoc test (*n* = 5 per group, * *p* < 0.05, ** *p* < 0.01, *** *p* < 0.001 vs. CON; ^#^ *p* < 0.05, ^##^ *p* < 0.01, ^###^ *p* < 0.001 vs. DSS; ^&^ *p* < 0.05, ^&&^ *p* < 0.01 vs. DSS + BA (50:50)). Detailed statistical differences were provided in Supplementary Figure S1.

First, we investigated the potential therapeutic effect of BS and AC (BA) extract mixtures in the DSS-induced colitis model. DSS, a chemical colitogen that has anticoagulant properties and induces epithelial disruption and intestinal inflammation, was administered at 2.5%, with or without various mixing ratios of BS:AC extract mixtures (0:100, 30:70, 50:50, 70:30, 100:0, 250 mg/kg/day; p.o.) for 5 days to prevent colitis. As shown in Figure 1, DSS treatment resulted in a decrease in body weight, a significant increase in stool constancy, and bloody stool, which were scored from 0 to 4 to obtain the DAI scores (Table 1), compared with the control group. In contrast, treatment with BA extracts mixture (50:50) alleviated body weight loss, stool constancy, bloody stool, and DAI scores during the DSS-induced colitis group (Figure 1A–D and Figure S1).

### 3.2. The Combined Ratio of BA Extracts Ameliorates Colon Shortening in the DSS-Induced Colitis Model

**Figure 2:**
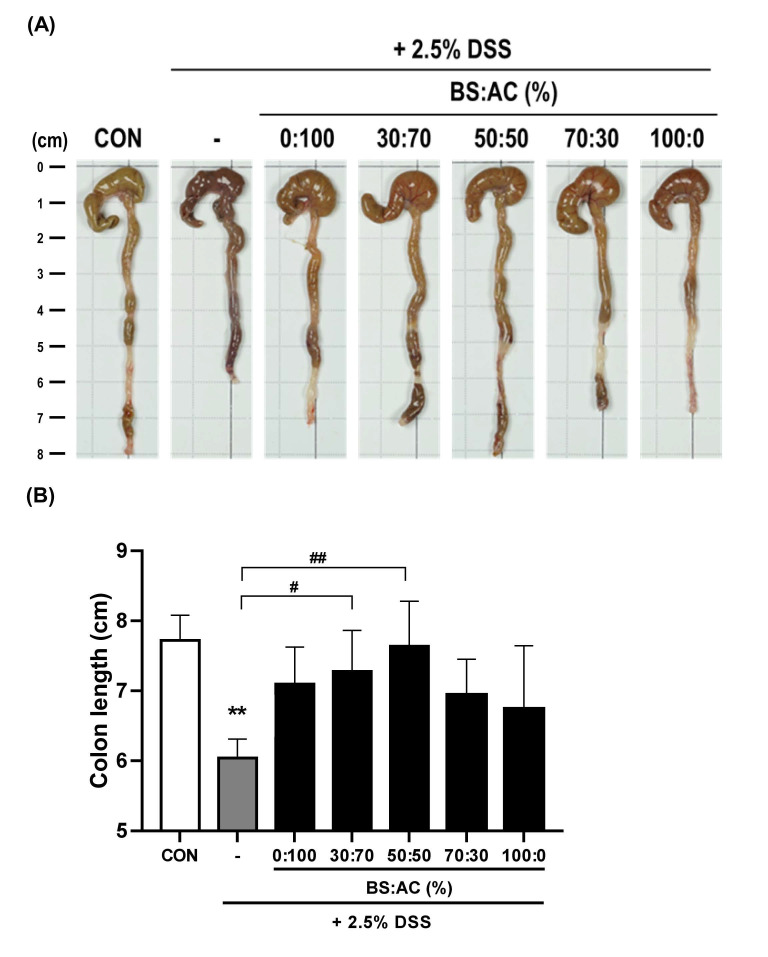
Effects of the ratio of BA extract mixtures on colon shortening in DSS-induced colitis. (**A**,**B**) A representative photograph of colon tissues in each group is shown. The data represent the percentage or mean ± SD. Statistical differences were determined by one-way ANOVA with Tukey’s post-hoc test (*n* = 5 per group, ** *p* < 0.01 vs. CON; ^#^ *p* < 0.05, ^##^ *p* < 0.01 vs. DSS).

Colon length shortening is a biological marker for colonic inflammation in the DSS-induced colitis model [30]. Thus, we examined whether oral administration of BA prevented colonic shortening in the DSS-induced colitis model by measuring the colon length in experimental mice. We observed that the colon length of the DSS-treated group (6.04 ± 0.27 cm, ** *p* = 0.0014 compare with the control group) was shorter than the control group (7.72 ± 0.36 cm). In contrast, treatment with BA (30:70, 7.28 ± 0.58 cm, ^#^ *p* = 0.029, compare with DSS-treated group) and BA (50:50, 7.64 ± 0.64 cm, ^##^ *p* = 0.0024, compare with DSS-treated group) resulted in longer colon length than in the DSS-treated group. Moreover, we found that treatment with BA (0:100, 7.1 ± 0.52 cm, *p* = 0.087) and BA (100:0, 6.74 ± 0.90 cm, *p* = 0.482) resulted in no significant difference in colon length than in the DSS-treated group. These data suggest that BA (30:70 and 50:50) treatment may alleviate DSS-induced colitis (Figure 2A,B).

### 3.3. The BA Extracts Ameliorate Histological Score in the DSS-Induced Colitis Model

**Figure 3:**
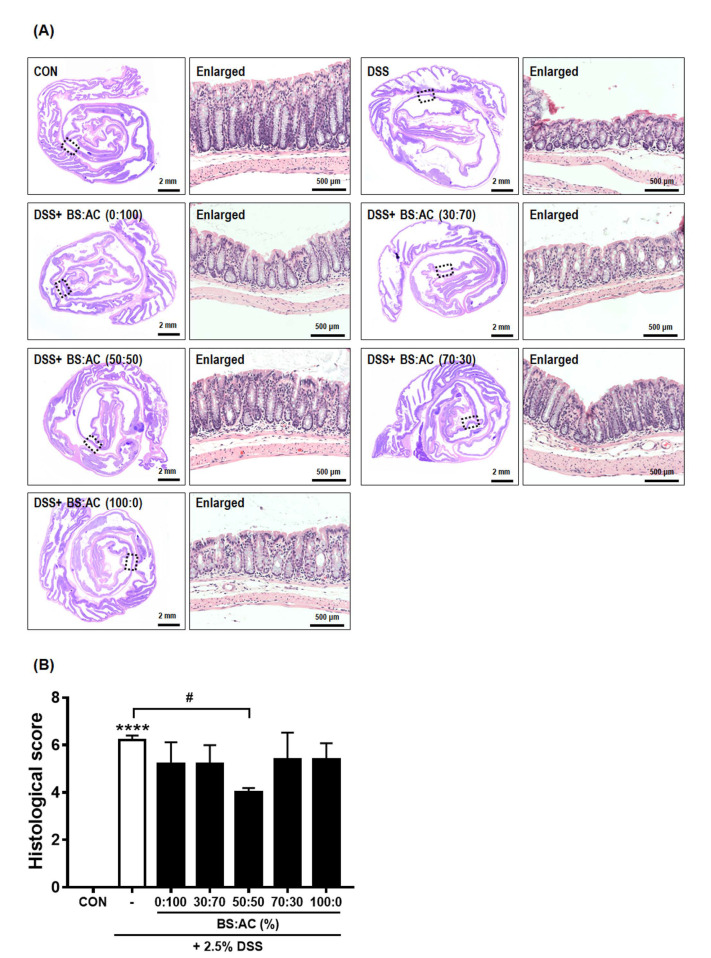
Effects of the ratio of BA extract mixtures on the histological score in DSS-induced colitis. (**A**) Representative images of Swiss roll mounts of colons were stained with H&E (**left panels**). Enlarged images (**right panels**) show magnified views of the boxed areas in the left panels. (**B**) Histological score in DSS-induced mice was estimated. Values are the mean ± SD (*n* = 5–6 per group, **** *p* < 0.0001 vs. CON; ^#^ *p* < 0.05 vs. DSS) significances were determined using one-way ANOVA with Tukey’s post-hoc test.

Colonic inflammation is accompanied by colonic mucosal disruption and ulceration, resulting in the infiltration of inflammatory monocytes and macrophages and the thickening of the lamina propria [31,32]. Therefore, we investigated the protective effect of BA in mucosal inflammation by H&E staining. As shown in Figure 3A, histological alterations in the colon of DSS-treated mice (5.6 ± 0.54, **** *p* < 0.0001, compared with the control group) have observed crypt distortion and inflammatory reactions such as mucosal and submucosal infiltrations compared with control mice. Interestingly, the BS:AC (50:50)-treated group (3.66 ± 1.86, ^#^ *p* < 0.05, compared with the DSS-treated group) significantly attenuated disruption of the architecture of colonic mucosa and ulceration compared to the DSS-treated group. However, we observed that treatment with BS:AC (0:100; 4.1 ± 1.34, *p* = 0.488), BS:AC (30:70, 4.8 ± 0.44, *p* = 0.857), BS:AC (70:30, 4.4 ± 0,54, *p* = 0.488), BS:AC (100:0, 4.8 ± 0.44, *p* = 0.857) resulted in no significant difference in colon length than in the DSS-treated group (Figure 3B).

### 3.4. Treatment with BA Attenuated the Protein Expression of Pro-Inflammatory Mediators (STAT3, COX2, iNOS, and NF-κB) in DSS-Induced Colitis

**Figure 4:**
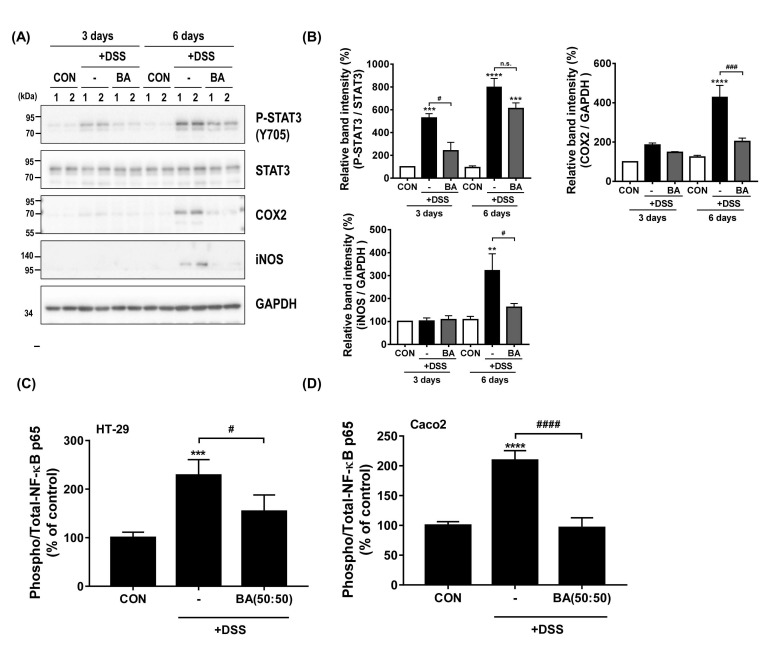
Effect of BA on pro-inflammatory mediator expression DSS-induced colitis. (**A**) Protein expression of pro-inflammatory mediators (P-STAT3, COX2, and iNOS) in mice colon from control, 2.5% DSS-treated, and 2.5% DSS with 250 mg/kg/day BA (BS:AC, 50:50) groups on 3 or 6 days. (**B**) The intensities of the bands in each panel were quantified for each group, and the values are expressed as the mean ± SD of three independent experiments. The data were obtained from three mice from each group. (**C**,**D**) ELISA using either phosphorylated-NF-κB p65 or total-NF-κB p65 antibody was used to measure the ratio of phospho-NF-κB p65/total-NF-κB p65 in HT-29 and Caco2 cells (control, 2% DSS-treated, and 2% DSS-treated with 200 μg/mL BA (BS:AC, 50:50) for 24 h). The phosphorylation levels were normalized against the control group and presented as the % of control. Statistical differences were determined by one-way ANOVA with Tukey’s post-hoc test (*n* = 3 per group, n.s.: not significant; ** *p* < 0.01, *** *p* < 0.001, **** *p* < 0.0001 vs. CON; ^#^ *p* < 0.05, ^###^ *p* < 0.001, ^####^ *p* < 0.0001 vs. DSS).

Previous studies have reported that the upregulation of inflammation-related proteins, including COX2, iNOS, NF-κB, signal transducer and activator of transcription (STAT) family, and activator protein 1 (AP-1), are associated with the immune deregulation of in vitro and in vivo colitis model [33,34]. Thus, we examined the anti-inflammatory effect of BA (50:50) treatment in the DSS-induced in vitro and in vivo colitis model using Western blot analysis and ELISA assay. As shown in Figure 4A,B, the DSS-treated group showed markedly increased phosphorylation of STAT3 (Tyr705) and COX2 and iNOS expression in the DSS-treated group. The expression of these proteins was markedly reduced by BA treatment (Figure 4B). In addition, we confirmed the effect of BA on NF-κB activation in DSS-treated cell lines. As shown in Figure 4C,D, incubation with BA markedly suppressed the phosphorylation of NF-κB (p65) in DSS-treated HT-29 and Caco2 cells (^#^ *p* < 0.05 and ^####^ *p* < 0.0001, compare with DSS group). These findings suggested that BA (50:50) treatment induces efficient attenuation of NF-κB and STAT3 activation, thereby affecting its regulatory proteins, including COX2 and iNOS.

### 3.5. Treatment with BA Reduces ROS Generation in DSS-Treated Human Colorectal Adenocarcinoma Cells

**Figure 5:**
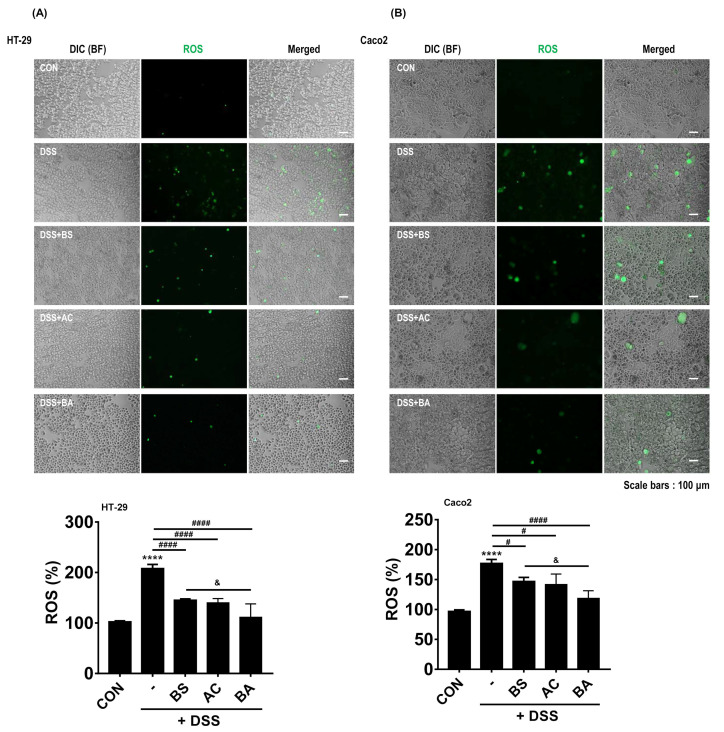
Effect of BA on ROS generation in DSS-treated cells. (**A**,**B**) Reduced ROS generation was shown by DCFH-DA staining in 2% DSS-treated HT-29 and Ca-co2 cells incubated with or without 200 μg/mL BS (BS:AC, 100:0), AC (BS:AC, 0:100), and BA (BS:AC, 50:50) analyzed by fluorescence microscopy (upper panels). All pictures are representative of multiple images from three independent experiments (scale bars: 100 μm). Quantification of ROS level in percentage DCFH-DA fluorescence in DSS-treated HT-29 and Caco2 cells incubated with 200 μg/mL BS, AC, and BA for 24 h. Fluorescence levels were performed as described (Section 2.10). The bar graph illustrates ROS generation normalized to and statistically compared to each group (bottom panels). Statistical differences were determined by one-way ANOVA with Tukey’s post-hoc test (*n* = 3 per group, **** *p* < 0.0001 vs. CON; ^#^ *p* < 0.05, ^####^ *p* < 0.0001 vs. DSS; ^&^ *p* < 0.05 vs. BS).

ROS overproduction is involved in the progression and pathogenesis of inflammatory diseases [35]. Evidence suggests that bamboo and AC extract have antioxidant activity [36,37,38]. Therefore, we evaluated the levels of ROS generation in DSS-treated human colorectal adenocarcinoma cell lines (HT-29 and Caco2) using the fluorescent DCFH-DA probe. We observed that DSS-treated HT-29 and Caco2 cells showed markedly increased ROS generation. In contrast, BA treatment [100:0 (BS), 0:100 (AC), and 50:50 (BA)] attenuated this ROS generation in DSS-treated HT-29 and Caco2 cells (Figure 5A). To confirm this finding, we measured the changes in ROS generation using a microplate spectrofluorometer. Interestingly, treatment with BA (50:50, HT-29: 111.32 ± 26.52%, ^####^ *p* < 0.0001; Caco2: 118.56 ± 12.76%, ^####^ *p* < 0.0001, compare with DSS group) more reduced ROS generation compared to 100:0 (BS, HT-29: 145.10 ± 6.53%, ^####^ *p* < 0.0001; Caco2: 146.73 ± 16.74%, ^#^ *p* < 0.05, compare with DSS group) and 0:100 (AC, HT-29: 139.92 ± 8.38%, ^####^ *p* < 0.0001; Caco2: 141.65 ± 17.61%, ^#^ *p* < 0.05, compare with DSS group) treatment in DSS-treated cells (HT-29: 207.79 ± 36.41%, Caco2: 176.99 ± 26.43%, **** *p* < 0.0001, compare with control group). We found that the BA treatment significantly decreased compared to BS treatment in DSS-treated HT-29 and Caco2 cells (*p* < 0.05, compared with the BS group) (Figure 5B).

### 3.6. Treatment with BA Decreases F-Actin Formation and RhoA Activity in DSS-Treated Human Colorectal Adenocarcinoma Cells

**Figure 6:**
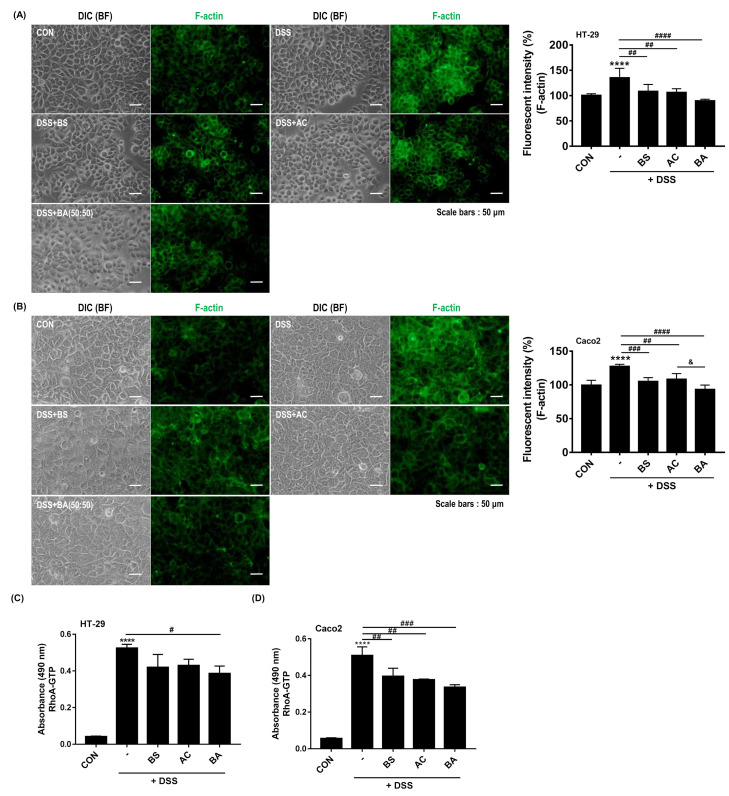
Effect of BA on F-actin formation and RhoA activity in DSS-treated cell lines. (**A**,**B**) Immunocytochemical staining for F-actin in 2% DSS-treated HT-29 and Caco2 cells with or without 200 μg/mL BS (BS:AC, 100:0), AC (BS:AC, 0:100), and BA (BS:AC, 50:50) for 24 h. Cells were fixed with 4% paraformaldehyde (PFA) and permeabilized with 0.2% Triton X-100 in PBS. F-actin was stained with Alexa Fluor 488-phalloidin. All pictures are representative of multiple images from three independent experiments (scale bars: 50 μm). Measurement of F-actin level performed as described (Section 2.11). Bar graph illustrates F-actin formation normalized to and statistically compared to each group (*n* = 5–8 per group **** *p* < 0.0001 vs. CON; ^##^ *p* < 0.01, ^###^ *p* < 0.001, ^####^ *p* < 0.0001 vs. DSS; ^&^ *p* < 0.05 vs. AC, right panels). (**C**,**D**) Measurement of RhoA-GTP by G-LISA RhoA activation assay in DSS-treated cells with or without BS, AC, and BA. The values were expressed as the mean ± SD of three independent experiments. Statistical differences were determined by one-way ANOVA with Tukey’s post-hoc test (*n* = 3 per group, **** *p* < 0.0001 vs. CON; ^#^ *p* < 0.05, ^##^ *p* < 0.01, ^###^ *p* < 0.001 vs. DSS).

RhoGTPases (RhoA, Rac1, and Cdc42) are key modulators of intestinal mucosa and GI diseases [39]. The RhoA signaling pathway is involved in the mechanism of IBD [25]. Particularly, RhoA activity regulates the cytoskeleton rearrangement through filamentous actin (F-actin) formation and motility [18,40,41]. Thus, we investigated the effect of BS (BS:AC, 100:0), AC (BS:AC, 0:100), and BA (BS:AC, 50:50) on F-actin formation in DSS-treated HT-29 and Caco2 cells. As shown in Figure 6A,B, F-actin formation was strongly observed in DSS-treated cells compared to untreated cells while treatment with BS, AC, and BS reduced F-actin formation compared to DSS-treated HT-29 and Caco2 cells. To confirm this finding, we measured the changes in F-actin levels in control, DSS-, and DSS with BS, AC, and BS-treated cells using a microplate spectrofluorometer. Consistent with the results of F-actin formation, DSS treatment resulted in a significant increase in F-actin formation (**** *p* < 0.0001, compare with control group), while treatment with BS (HT-29: 108.32 ± 13.73%, ^##^ *p* < 0.01; Caco2: 105.11 ± 5.66%, ^###^ *p* < 0.001), AC (HT-29: 106.18 ± 7.46%, ^##^ *p* < 0.01; Caco2: 108.25 ± 8.50%, ^##^ *p* < 0.01) and BA (HT-29: 89.64 ± 3.21%, ^####^ *p* < 0.0001; Caco2: 93.15 ± 6.61%, ^####^ *p* < 0.0001) significantly decreased F-actin formation compared to DSS-treated group. Furthermore, we confirmed that BA treatment significantly reduced F-actin formation compared to AC treatment in DSS-treated Caco2 cells (^&^ *p* < 0.05, Figure 6B). In addition, to elucidate whether F-actin formation regulated by BA extracts mixtures treatment was due to RhoA activity, we used the RhoA G-LISA activation assay, a quantitative method for evaluating RhoA-guanosine triphosphate (GTP) levels. Treatment with BA(HT-29: 0.38 ± 0.04, ^#^ *p* < 0.05, Caco2: 0.33 ± 0.01, ^###^ *p* < 0.001, compare with DSS group) more attenuated RhoA activation than BS (HT-29: 0.41 ± 0.07; Caco2: 0.39 ± 0.04) and AC (HT-29: 0.42 ± 0.03, ^##^ *p* < 0.01; Caco2: 0.37 ± 0.004, ^##^ *p* < 0.01, compare with DSS group) in DSS-treated HT-29 and Caco2 cells (HT-29: 0.52 ± 0.02; Caco2: 0.50 ± 0.04, **** *p* < 0.0001, compare with control group) (Figure 6C,D). These findings suggest that BA treatment is involved in F-actin formation by regulating RhoA activity in DSS-induced colitis.

## 4. Discussion

This study demonstrated the therapeutic effect of BS and AC (BA) extract mixture in DSS-induced colitis. BA extract mixture leads to protection from colonic inflammation, disruption of the architecture of colonic mucosa, and ulceration. These therapeutic effects were mediated by inhibiting the NF-κB-STAT3 signaling pathway and RhoA/Rho-associated kinase (ROCK) activity, which controls colonic inflammation, F-actin polymerization, and plays an important role in the pathogenesis of colitis.

NF-κB and STAT3 signaling pathways regulate gene transcription to influence the pathological evolution of inflammatory and immune diseases [42,43,44]. Numerous studies have shown that NF-κB-STAT3 activation plays a key role in the pathogenic development of ulcerative colitis [32,43,45,46]. In the DSS-induced colitis model, inhibition of NF-κB and STAT3 activities ameliorated colonic inflammation by downregulating pro-inflammatory proteins [45]. In our data, BA treatment decreased the NF-κB and STAT3 activities and the expression of COX2 and iNOS in the DSS-treated mice model (Figure 4), which provides evidence of how BA has an anti-inflammatory effect in DSS-induced colitis.

The dysregulation of RhoA/ROCK activity in the intestinal epithelium is associated with cytoskeletal dysfunction [47], and the inhibition of RhoA/ROCK protects against DSS- or TNBS-induced colitis in mice by attenuating intestinal epithelial barrier dysfunction [23,48] mediated by the NF-κB-STAT3 signaling pathway [49]. Collectively, these reports suggested that RhoA/ROCK activity plays an important role in the pathogenesis of colitis and colonic inflammation. Intriguingly, our study demonstrated that BA (BS:AC, 50:50) treatment significantly decreased the level of RhoA-GTP (an active form of RhoA) more than single extracts in DSS-treated cells (Figure 6). In addition, recent studies have demonstrated a functional link between RhoA/ROCK and STAT3 signaling pathways. RhoA activity modulated the STAT3 activation partly depending on ROCK. Moreover, ROCK2 interacts with phosphorylated-STAT3, and its kinase activity controls the formation of the ROCK2/STAT3/JAK2 complex [50]. These data indicated that RhoA/ROCK-STAT3 signaling pathway is involved in colonic inflammation and pathogenesis of colitis

In addition, the balance of the actin filament is important for regulating actin cytoskeletal dynamics and has been associated with cell contraction, adhesion, migration, and division. Moreover, changes in F-actin formation in the intestinal epithelial cytoskeleton are related to intestinal barrier damage [51]. A recent experimental study reported that the deficiency of cortactin (a regulator of the intestinal epithelial barrier) increased the RhoA/ROCK1-dependent actomyosin contractility, and ROCK1 inhibition rescued the barrier defect [48]. Moreover, cofilin (an actin-binding protein) promotes actin dynamics through the RhoA- ROCK-LIMK (LIMK kinase)-cofilin signaling pathway, inducing the polymerization of actin filaments [52]. RhoA/ROCK activation abolishes the actin-binding activity of cofilin by inducing cofilin phosphorylation (Ser 3), resulting in enhanced actin filament polymerization [53]. Our study also found that DSS treatment increased the RhoA-GTP levels and induced F-actin polymerization in HT-29 and Caco-2 cells. In contrast, treatment with BA significantly decreased this phenomenon. These observations showed that BA treatment attenuated altered F-actin formation by inhibiting RhoA/ROCK activity in DSS-treated cells. Consequently, these findings suggest that BA treatment may play an important role in DSS-induced colitis by regulating RhoA/ROCK activity. However, previous studies reported that the RhoA-ROCK-LIMK-cofilin signaling pathway is involved in the fibrosis of various organs [54,55,56,57]. However, the exact regulatory mechanism of intestinal fibrosis has not been elucidated. Therefore, it is necessary to explore intestinal fibrosis in colitis models further.

The imbalance between generations of ROS is involved in the progression of various inflammatory disorders, including those involving the gastrointestinal (GI) tract [58], through altering the antioxidant defense system, leading to excess oxidative stress production [59]. Furthermore, recent studies reported that increased oxidative stress had been associated with autoimmune disorders, including rheumatoid arthritis, systemic lupus erythematosus, psoriasis, and celiac disease; and neurodegenerative diseases such as Alzheimer’s and Parkinson’s disease, amyotrophic lateral sclerosis, and multiple sclerosis [60]. Oxidative stress has been shown to cause gastroduodenal ulceration [61], IBD [62], and even gastric and colorectal cancer. Interestingly, our data demonstrated that DSS-treated cells showed markedly increased ROS generation. In contrast, BA treatment attenuated ROS overproduction in DSS-treated HT-29 and Caco2 cells (Figure 5). These results suggest that BA treatment attenuated the imbalance of ROS generation and excess oxidative stress that may be associated with the progression of pathological changes observed in colonic inflammation.

Taken together, our results showed that the BA extract mixture has a therapeutic role in colitis that contributes to reducing colonic inflammation by decrement NF-κB-STAT3 phosphorylation and RhoA/ROCK activity, which leads to attenuated disruption of the architecture of colonic mucosa and ulceration. These findings are important for understanding the protective mechanisms of DSS-induced colitis and its mediated signaling pathway.

## 5. Conclusions

Our results demonstrated a synergistic protective effect of BS and AC extracts. BS and AC extract mixture (BA, 50:50) could be protected with higher efficacy against colonic inflammation and disruption of the architecture of colonic mucosa and ulceration in DSS-induced colitis compare with single extract treatment. These findings are important for the prevention and treatment of IBD using natural products, which can alleviate symptoms of IBD and be effective, less toxic, and cost-effective.

## Figures and Tables

**Table 1:** Disease activity index (DAI) scoring.

Disease Activity Index (DAI)			
Score	Weight Loss	Stool Consistency	Bleeding
0	None	Normal	Normal
1	0–10%	Soft but still formed	
2	10–15%	Loose stools	Hemocult+
3	15–20%		
4	>20%	Diarrhea	Gross Bleeding

The DAI score was calculated as the sum of scores of % weight loss, stool consistency and blood in feces. Normal stools: pellets shape; loose: pasty stools, which do not stick to the anus; diarrhea: liquid stools that stick to the anus.

## Data Availability

Not applicable.

## Supplementary Materials

The following supporting information can be downloaded at: https://www.mdpi.com/article/10.3390/cimb44100345/s1, Figure S1: Effects of the ratio of BA extract mixtures on body weight loss, stool constancy, bloody stool, and DAI score.
