# Supplementary material for: Bamboo Shoot and Artemisia capillaris Extract Mixture Ameliorates Dextran Sodium Sulfate-Induced Colitis
Source: Curr Issues Mol Biol. 2022 Oct 20;44(10):5086–103. doi: 10.3390/cimb44100345 (PMC9600592; doi:10.3390/cimb44100345)
Supplement: Supplementary file 1 [file cimb-44-00345-s001.zip › cimb-1933472-supplementary.pdf]

Supplementary Figure S1. Effects of the ratio of BA extract mixtures on body weight loss, stool constancy, bloody stool, and DAI score

**DAY**	**2 day**				**3 day**			
**Group**	**(%)**	**score**			**(%)**	**score**		
	**Weight**	**stool consistency**	**bloody stool**	**DAI**	**Weight(%)**	**stool consistency**	**bloody stool**	**DAI**
**CON**	100.67±0.58	0±0	0±0	0±0	102.77±2.58	0±0	0±0	0.2±0.44
**2.5% DSS**	98.87±2.02	0±0	0.8±1.09	1.6±1.34	99.51±2.79	1.0±0, *	1.6±0.89, *	3.0±1.0, **
**DSS+BA (BS:AC,100:0)**	100.34±0.82	0.2±0.44	0±0	0.25±0.5	100.95±1.74	0.2±0.44	0.8±1.09	1.75±1.25
**DSS+BA (BS:AC, 70:30)**	100.16±0.55	0±0	0±0	0.4±0.54	99.59±1.26	0.8±0.44	0.4±0.89	1.8±1.09
**DSS+BA (BS:AC, 50:50)**	100.26±1.59	0±0	0±0	0.6±0.54	100.09±0.54	0.2±0.44	0.4±0.89	0.6±0.89, $^{\#\#}$
**DSS+BA (BS:AC, 30:70)**	100.01±2.29	0.2±0.44	0.4±0.89	1.2±1.30	100.60±2.39	0.6±0.54	0.8±1.09	1.8±2.04
**DSS+BA (BS:AC, 0:100)**	101.75±4.18	0.6±0.54	0.8±1.09	1.8±1.09	102.42±3.68	0.4±0.54	1.2±1.09	1.8±1.30

**DAY**	**4 day**				**5 day**			
**Group**	**(%)**	**score**			**(%)**	**score**		
	**Weight**	**stool consistency**	**bloody stool**	**DAI**	**Weight(%)**	**stool consistency**	**bloody stool**	**DAI**
**CON**	99.98±2.74	0±0	0±0	0.2±0.44	103.14±2.97	0±0	0±0	0±0
**2.5% DSS**	99.66±3.58	1.0±0.7, *	3.6±0.89, ***	5.0±1.41, ***	96.72±4.66, ***	2.8±1.09, ***	4.0±0, ***	7.6±1.14, ***
**DSS+BA (BS:AC,100:0)**	99.93±0.98	0.8±0.44	2.0±2.0, $^{**,\#}$	3.75±1.5′ ***	97.29±2.37, **	2.0±0, ***	3.6±0.89, ***	6.4±1.34, ***
**DSS+BA (BS:AC, 70:30)**	98.76±1.83	1.4±1.51, $^{**,\&\&}$	2.0±1.41, $^{**,\#}$	4.0±2.12, $^{***,\&}$	96.95±2.78, ***	1.4±0.89, $^{**,\#\#}$	4.0±0, ***	6.2±1.09, ***
**DSS+BA (BS:AC, 50:50)**	101.21±1.93	0.2±0.44	1.2±1.09, $^{\#\#\#}$	2.0±0.70, $^{\#\#\#}$	99.91±2.32	1.8±1.30, $^{***,\#}$	4.0±0, ***	5.4±0.89, $^{***,\#}$
**DSS+BA (BS:AC, 30:70)**	99.67±2.24	1.4±0.54, $^{**,\&\&}$	2.4±1.67, ***	4.6±2.07, $^{***,\&\&}$	98.09±4.12, *	1.8±1.30, , $^{***,\#}$	3.2±1.09, ***	5.8±2.38, ***
**DSS+BA (BS:AC, 0:100)**	100.75±6.00	1.2±0.83, $^{**,\&}$	2.0±2.0, $^{**,\#}$	3.4±2.07, ***	100.32±5.35	1.4±0.54, , $^{***,\#\#}$	3.2±1.09, ***	5.2.±1.78, $^{***,\#}$

Data are mean ± SD (n = 5 per group)

***** $p < 0.05$; ** $p < 0.01$; *** $p < 0.001$ **compared to control group**

$^{\#}p < 0.05$; $^{\#\#}p < 0.01$; $^{\#\#\#}p < 0.001$ compared to 2.5% DSS group

$^{\&}p < 0.05$; $^{\&\&}p < 0.01$ compared to DSS+BA (BS:AC, 50:50) group
